# Dissemination and implementation of suicide prevention training in one Scottish region

**DOI:** 10.1186/1472-6963-8-246

**Published:** 2008-12-03

**Authors:** Linda Gask, Gillian Lever-Green, Rebecca Hays

**Affiliations:** 1 School of Community-based Medicine, University of Manchester, NPCRDC, 5th Floor, Williamson Building, Oxford Road, Manchester, M13 9PL, UK

## Abstract

**Background:**

As part of a national co-ordinated and multifaceted response to the excess suicide rate, the *Choose Life *initiative, the Highland Choose Life Group launched an ambitious programme of training for National Health Service (NHS), Council and voluntary organisation staff. In this study of the dissemination and implementation of STORM (Skills-based Training On Risk Management), we set out to explore not only the outcomes of training, but key factors involved in the processes of diffusion, dissemination and implementation of the educational intervention.

**Methods:**

Participants attending STORM training in Highland Region provided by 12 trained facilitators during the period March 2004 to February 2005 were recruited. Quantitative data collection from participants took place at three time points; immediately before training, immediately post-training and six months after training. Semi-structured telephone interviews were carried out with the training facilitators and with a sample of course participants 6 months after they had been trained. We have utilized the conceptual model described by Greenhalgh and colleagues in a Framework analysis of the data, for considering the determinants of diffusion, dissemination and implementation of interventions in health service delivery and organization.

**Results:**

Some 203 individuals completed a series of questionnaire measures immediately pre (time 1) and immediately post (time 2) training and there were significant improvements in attitudes and confidence of participants. Key factors in the diffusion, dissemination and implementation process were the presence of a champion or local opinion leader who supported and directed the intervention, local adaptation of the materials, commissioning of a group of facilitators who were provided with financial and administrative support, dedicated time to provide the training and regular peer-support.

**Conclusion:**

Features that contributed to the success of STORM were *related to both the context *(the multi-dimensional support provided from the host organisation and the favourable policy environment) *and the intervention *(openness to local adaptation, clinical relevance and utility), and the dynamic interaction between context and the intervention.

## Background

Across Scotland during the period 1989–2004 male suicide rates increased by 22 percent and female suicide rates by 6 percent [1]. Over this period, Scotland has experienced a greater number of deaths from suicide than other countries in the UK [2] and the increase in deaths in men has led to a change in its rank from 12^th ^to 11^th ^highest rate within Europe [3].

Highland Region has one of the highest rates of suicide in Scotland [4,5]. Deaths by drowning, firearms and by others gases and vapours (mainly due to car exhausts) are overrepresented in the Highlands in comparison with the rest of Scotland. The reasons for these higher rates in the Highlands are unknown, but a number of factors could be involved. Many people who attempt to harm themselves are under the influence of alcohol and misuse of alcohol is a common problem in Scotland [6]. NHS Highland's catchment area comprises the largest and most sparsely populated part of the UK  and there are barriers to accessing mental health care in rural areas including distance from services and a fear of being identified as accessing services [7]. Additionally, the excess suicide rate in farmers is well recognised [8]. Farmers have access to lethal means including firearms [9] and may differ in their help-seeking behaviour, with more presentation of physical symptoms of emotional distress [10].

As part of a national co-ordinated and multifaceted response to the excess suicide rate, the *Choose Life *initiative [11], the Highland Choose Life Group launched an ambitious programme of training for National Health Service (NHS), Council and voluntary organisation staff. Training for health workers has been advocated as a key route to suicide prevention in a number of countries [12]. However, reports of evaluation of staff training interventions are relatively scarce in the literature [13-17].

STORM (Skills-based Training On Risk Management) is a package originally developed by the University of Manchester. The content of the intervention reflects established assessment and management methods for patients with suicidal ideation and/or feelings of hopelessness [18-21]. STORM uses a handbook to provide background knowledge, and to reinforce and remind participants of the skills that are the main focus of the training.

The format of the intervention derives from adult learning theory [22], Bandura's Social Learning Theory [23] and published literature on changing the behaviour of health care workers [24]. STORM is primarily concerned with developing complex clinical communication skills and so utilizes role-play and video-feedback on performance. Attitudes also need to be addressed, which requires interactive self reflection and reflection on the experiences of peers and case material demonstrated on videotape. If participants' current beliefs are challenged and changed in an interactive learning setting, then, with practise, they may also change their practice to reflect the change in belief (otherwise they will encounter cognitive dissonance, usually experienced as anxiety or frustration).

We have carried out three previous evaluations of STORM training and have demonstrated that the intervention brings about a change in skills and attitudes of participants [25-27]. We have also demonstrated that the training can be delivered to a multidisciplinary, multi-professional audience across a single health district in England [26], but this intervention did not result in a change in the suicide rate [28]. Therefore, we hypothesized that training was likely to be a necessary, but not sufficient component of a comprehensive multi-faceted initiative. In a further study with mental health professionals, we examined the process and outcome of implementation of suicide prevention training in specialist mental health trusts in England and raised concerns about the lack of an educational culture within these organisations that might foster acquisition of new skills [27]. In this study of the diffusion, dissemination and implementation of STORM training in the Highland Region of Scotland, we aimed not only to replicate the key features of our original English study of community delivery of training [26] but to explore in detail the processes involved in the uptake of the educational intervention; Working definitions in this paper are summarised by Greenhalgh and colleagues [29]: diffusion is *'a passive phenomenon of social influence' *whilst dissemination is defined as *'active and planned efforts to persuade target groups to adopt an innovation'*. Adoption of an intervention, either by individuals or an organization, is underpinned by both of these processes. Implementation is *the 'active and planned efforts to mainstream an innovation within an organisation'*. Implementation incorporates the concept of 'sustainability' (known also as routinization and institutionalisation). It could be argued that implementation is the embodiment of all these processes but for simplicity we have here dealt with each separately.

## Methods

### Data and study sample

Participants attending STORM training in Highland Region provided by 12 trained facilitators during the period March 2004 to February 2005 were recruited. The trainers came from mental health services, and the majority of these were nurses, however others trained included psychologists, social workers, managers and a service user. Data collection from participants took place at three time points; immediately before training, immediately post-training and six months after training (see table 1). Written consent was obtained from all participants. The immediately pre and immediately post training questionnaires were handed out by facilitators within the training, but the six month questionnaires were sent out by post; if no response was received within a month, a second copy of the questionnaire was sent out and telephone contact was attempted to remind participants about the questionnaire. Unlike in our original study of training, we did not seek to collect any pre- and post-training videotaped recordings of participants role-play, as this would have been logistically difficult given the distance between Manchester and the Highland region, with no on-site researcher.

**Table 1:** Timetable of assessments

**Time**	**Assessments**
**Baseline**	Attitudes to Suicide Prevention Scale Visual analogue confidence scale

**Immediately post-training**	Attitudes to Suicide Prevention Scale Visual analogue confidence scale Satisfaction questionnaire

**Six months post-training**	Attitudes to Suicide Prevention Scale Visual analogue confidence scale Impact on clinical practice questionnaire Selected sample only: semi-structured interview

We asked participants about age, gender, ethnicity, number of years in profession and previous suicide prevention training. Our main quantitative outcome measures were change in attitude to suicide prevention and confidence in the management of suicidal patients/clients. Attitudes were rated using the 'Attitudes to Suicide Prevention (ASP) Scale', where lower scores indicate more positive attitudes [30]. Confidence in the assessment and management of suicidal patients/clients was measured using a 100 mm visual analogue scale developed for the first STORM study. A minimum score of '0' is rated as 'not at all confident', while a maximum score of '100' is rated as 'very confident'. Satisfaction with participation in training was assessed using a questionnaire developed for our previous studies, focusing on satisfaction with specific aspects of the training package. Impact on clinical practice was assessed by an open-ended written questionnaire in which we asked participants to provide comments about how each of the training modules had been of use in their everyday work, All of the above areas were also addressed in the semi-structured telephone interviews with a sample of course participants and with a sample of course participants 6 months after they had been trained. A purposive sample of 12 participants was recruited on the basis of confidence and attitude scores (high or low initial scores and no change, high or low change scores), gender, age, previous training and profession to obtain a sample with maximum variation but that was also representative of the group as a whole.

In order to explore the processes of diffusion, dissemination and implementation in depth we carried out semi-structured telephone interviews with the training facilitators (10 of the 12 facilitators were interviewed between 6 and 24 months post training with 5 of the facilitators being interviewed on two separate occasions at least 7 months apart) see table 2 for interview schedules and table 3 for details of interviewees). We also interviewed the Consultant in Public Health (Dr Cameron Stark) and the Choose Life Coordinator for Highland (Michael Perera). All interviews were recorded and transcribed.

**Table 2:** Semi-structured interview questions

**Participants**	**Interview questions**
**STORM training facilitators:**	1. What challenges, positive and negative, did you face whilst *delivering *training?
	2. What challenges, positive and negative, did you face whilst *organising *the training – logistical, staffing and/or managerial issues?
	3. Do you or the organisation have any concerns or issues with the delivery and/or translation of STORM?
	4. Have you or the organisation employed any strategies to help implement STORM and/or do you feel that there is a need for any strategies?
	5. How do you feel STORM was received generally (into practice, policy and culture)?
	6. What changes, if any, have been made as a direct consequence of STORM?

**STORM training participants:**	1. Tell me about your everyday working environment.
	2. Can you tell me your views on whether suicide can be prevented by the actions of others?
	3. What were your colleagues and managers attitudes toward you receiving STORM training?
	4. How did your personal attitude toward suicide change as a result of the STORM training?
	5. What training have you had in the past in assessing and managing suicide risk?
	- Which of the 4 STORM training modules did you receive?
	6. How has attending STORM training affected your everyday work?
	- Can you think of a specific case where you acted differently as a result of the STORM training? If so, in what way? What would you have done before?
	7. Are there any circumstances involving a suicidal patient where you think that you would act differently after having received the training?
	8. Has it been difficult to put elements of the course into practice? How? Why do you think that has happened?
	9. What was most (and least) about the training course?
	10. Whom were you trained alongside? How did you find that?
	11. How do you think STORM training could be improved (explore content and format issues)?
	12. Which professional groups do you think such training should be offered to?

**Table 3:** Details of course participants who were interviewed

**Code**	**Profession**	**Age**	**Gender**	**Previous training**
H25	GP	52	Male	No

H90	Social Worker	57	Male	Yes

H98	Occupational Therapist	48	Female	No

H103	Nurse	43	Male	Yes

H110	Nurse	43	Female	Yes

H119	Nurse	37	Female	Yes

H131	Nurse	-	Female	No

H142	Volunteer Worker	34	Female	No

H172	Nurse	28	Female	Yes

H159	Social Worker	26	Female	No

H191	Day Centre Officer	49	Male	No

H193	Social Worker	37	Male	Yes

### Ethical approval

This study was carried out as part of a multi-site evaluation of STORM training, which was approved by the North West Multi-Centre Research Ethics Committee MREC reference number: 04MRE08/8.

### Analysis

Quantitative data were analysed using SPSS version 13.0 (SPSS Inc., Chicago, IL, USA) with paired two-tailed t-tests for data that were normally distributed and Wilcoxon Signed Ranks tests for ordinal data. Statistical significance was set at the 5% level.

Qualitative data was subjected to a Framework analysis [31] within MAXQDA software. The conceptual model of the diffusion, dissemination and implementation of interventions in health service delivery, described by Greenhalgh and colleagues [29], as utilized in the development of a framework/coding schedule. A template coding schedule was developed collaboratively by all three authors, from an initial discussion of a sub-set of ten transcripts. Additionally, all authors participated in coding the transcripts and ongoing discussions, which led to revisions of and improvements to the template. The theory of routinization, described by Yin [32], was incorporated into the framework in order to understand how STORM training translates into practice and organizational systems.

## Results and discussion

### Attitudes, confidence and overall satisfaction

Some 203 individuals completed a series of questionnaire measures immediately pre (time 1) and immediately post (time 2) training during the period March 2004 to February 2005. Sixty (31%) individuals also returned follow-up questionnaires sent out six months post training (time 3); although disappointingly low, this figure is in the same range as our previous experience of following up health professionals [26].

Of the total sample, the mean age was 43, 73% were female, 60% were born in Scotland (27% in England) and 98% specified 'White' as their ethnic group. 64% had received no previous training on suicide risk assessment or prevention and for the majority of those who had received previous training (68%) the duration had been of eight hours or less.

Nurse (38%) and social worker (20%) were the most common professional groups to receive/take part in training with 50% of those trained working in adult mental health services. However, a wide range of health and social care professionals participated including support workers (19), doctors (16 including 3 psychiatrists), health visitors (5), occupational therapists (4), a housing officer, nursery nurses (2) and a police officer. They managed a full range of client groups including children and young adults.

As in our previous studies [25-27], there were significant improvements in attitude and confidence (see tables 4 to 7). Confidence scores increased significantly from time 1 to time 2 (table 6) and from time 1 to time 3 (table 7), for each of the four items; therefore, confidence levels improved during/with training and remained improved over a period of 6 months. For each of the fourteen attitude items, scores decreased from time 1 to time 2 (table 4); therefore attitudes became more positive with/during training. All but two items (3 and 7) also showed a decrease in attitude score from time 1 to time 3 (table 5); therefore attitudes remained more positive up to six months post training.

**Table 4:** Change in attitudes to suicide prevention: pre-training – immediately post-training.

**Item**		**N**	**Time 1 (Pre)**		**Time 2 (Post)**		**Mean change**		**P**
			**Mean**	**SD**	**Mean**	**SD**	**Mean**	95% CI*	value**
1	I resent being asked to do more about suicide.	201	1.50	0.63	1.43	0.54	-0.06	-0.13 – +0.00	0.077

2	Suicide prevention is not my responsibility.	202	1.70	0.70	1.56	0.61	-0.14	-0.24 – -0.04	0.005

3	Making more funds available to the appropriate health services would make no difference to the suicide rate.	202	2.04	0.76	1.88	0.89	-0.16	-0.28 – -0.04	0.003

4	Working with suicidal patients is rewarding.	202	2.54	0.77	2.27	0.70	-0.28	-0.39 – -0.17	0.000

5	If people are serious about committing suicide they don't tell anyone.	203	2.30	0.87	1.81	0.67	-0.49	-0.61 – -0.37	0.000

6	I feel defensive when people offer advice about suicide prevention.	199	1.95	0.73	1.82	0.64	-0.14	-0.25 – -0.02	0.014

7	It is easy for people not involved in clinical practice to make judgements about suicide prevention.	196	3.42	0.91	3.32	1.02	-0.10	-0.23 – +0.03	0.199

8	If a person survives a suicide attempt, then this was a ploy for attention.	202	1.86	0.73	1.53	0.58	-0.33	-0.42 – -0.23	0.000

9	People have the right to take their own lives.	196	3.28	0.90	3.26	0.86	-0.02	-0.11 – +0.07	0.601

10	Since unemployment & poverty are the main causes of suicide there is little that an individual can do to prevent it.	202	1.74	0.50	1.66	0.56	-0.08	-0.16 – -0.00	0.026

11	I don't feel comfortable assessing for suicide risk.	203	2.70	1.01	2.01	0.69	-0.69	-0.82 – -0.56	0.000

12	Suicide prevention measures are a drain on resources which would be more useful elsewhere.	201	1.72	0.63	1.51	0.52	-0.21	-0.30 – -0.13	0.000

13	There is no way of knowing who is going to commit suicide.	202	2.53	0.84	2.17	0.86	-0.37	-0.48 – -0.25	0.000

14	What proportion of suicides do you consider preventable?	196	2.70	0.75	2.46	0.70	-0.24	-0.33 – -0.15	0.000

	Total	180	32.06	4.63	28.69	4.58	-3.37	-3.93 – -2.81	0.000

Scoring range (per item):1 (positive attitude) – 5 (negative attitude)

*paired t-test **wilcoxon signed ranks test

**Table 5:** Change in attitudes to suicide prevention: pre-training – 6 months post-training.

**Item**		**N**	**Time 1 ** **(Pre)**		**Time 3 ** **(6 Month)**		**Mean ** **change**		**P**
			**Mean**	**SD**	**Mean**	**SD**	**Mean**	95% CI*	value**
1	I resent being asked to do more about suicide.	59	1.47	0.63	1.39	0.49	-0.08	-0.26 – +0.09	0.384

2	Suicide prevention is not my responsibility.	60	1.58	0.70	1.53	0.70	-0.05	-0.27 – +0.17	0.685

3	Making more funds available to the appropriate health services would make no difference to the suicide rate.	60	2.07	0.73	2.08	0.85	+0.02	-0.23 – +0.27	0.893

4	Working with suicidal patients is rewarding.	59	2.53	0.73	2.39	0.87	-0.14	-0.37 – +0.10	0.191

5	If people are serious about committing suicide they don't tell anyone.	60	2.23	0.74	2.05	0.89	-0.18	-0.39 – +0.02	0.079

6	I feel defensive when people offer advice about suicide prevention.	60	1.98	0.75	1.78	0.64	-0.20	-0.41 – +0.01	0.058

7	It is easy for people not involved in clinical practice to make judgements about suicide prevention.	59	3.17	0.87	3.34	0.90	+0.17	-0.13 – +0.47	0.311

8	If a person survives a suicide attempt, then this was a ploy for attention.	60	1.87	0.62	1.63	0.58	-0.23	-0.40 – -0.07	0.008

9	People have the right to take their own lives.	57	3.37	0.98	3.21	0.94	-0.16	-0.36 – +0.05	0.084

10	Since unemployment & poverty are the main causes of suicide there is little that an individual can do to prevent it.	60	1.78	0.49	1.68	0.54	-0.10	-0.26 – +0.06	0.221

11	I don't feel comfortable assessing for suicide risk.	60	2.67	1.13	1.97	0.78	-0.70	-1.00 – -0.40	0.000

12	Suicide prevention measures are a drain on resources which would be more useful elsewhere.	60	1.67	0.63	1.62	0.67	-0.05	-0.22 – +0.12	0.371

13	There is no way of knowing who is going to commit suicide.	60	2.40	0.76	2.08	0.72	-0.32	-0.48 – -0.15	0.000

14	What proportion of suicides do you consider preventable?	56	2.84	0.83	2.57	0.78	-0.27	-0.47 – -0.07	0.013

	Total	53	31.81	4.49	29.43	4.53	-2.38	-3.35 – -1.41	0.000

Scoring range (per item):1 (positive attitude) – 5 (negative attitude)

*paired t-test **wilcoxon signed ranks test

**Table 6:** Change in confidence: pre-training – immediately post-training.

**Item**		**N**	**Time 1 (Pre)**		**Time 2 (Post)**		**Mean change**		**P**
			**Mean**	**SD**	**Mean**	**SD**	**Mean**	95% CI*	value*
1	I am confident that I have the interview skills to use my time well with suicidal clients.	195	47.43	20.38	69.62	14.84	22.19	19.87 – 24.51	0.000

2	After seeing a client once I would be confident that I could recognise potential suicide risk.	195	35.35	19.93	63.82	17.92	28.46	25.79 – 31.13	0.000

3	I feel confident that I could differentiate a mild depression from a suicide risk.	195	49.74	22.72	69.66	17.34	19.92	17.45 – 22.39	0.000

4	I am confident in dealing with the needs of suicidal clients.	195	41.17	21.19	65.82	16.91	24.65	22.07 – 27.23	0.000

	Total	195	173.70	71.43	268.81	60.92	95.22	87.43 – 103.00	0.000

Scoring range (per item): 0 (not at all confident) – 100 (very confident)

*paired t test

**Table 7:** Change in confidence: pre-training – 6 months post-training.

**Item**		**N**	**Time 1 (Pre)**		**Time 3 (6 Month)**		Mean change*		P value*
			**Mean**	**SD**	**Mean**	**SD**	**Mean**	**95% CI**	
1	I am confident that I have the interview skills to use my time well with suicidal clients.	59	51.69	21.60	68.83	16.33	17.14	12.88 – 21.39	0.000

2	After seeing a client once I would be confident that I could recognise potential suicide risk.	59	37.44	21.33	58.05	20.07	20.61	14.96 – 26.26	0.000

3	I feel confident that I could differentiate a mild depression from a suicide risk.	59	49.95	23.14	68.37	16.20	18.42	13.29 – 23.56	0.000

4	I am confident in dealing with the needs of suicidal clients.	59	42.75	21.95	65.98	17.16	23.24	18.53 – 27.95	0.000

	Total	59	181.83	74.59	261.24	61.20	79.41	63.55 – 95.26	0.000

Scoring range (per item): 0 (not at all confident) – 100 (very confident)

*paired t test

Analysis of non-responders revealed that a higher proportion of males than females returned the time 3 questionnaire measures (42% compared with 26%) and a chi-squared test showed this to be a significant difference at the 5% level (p < 0.05). There were no differences in terms of age, previous training or profession but those who responded at time 3 had more years experience in their profession on average than those who did not respond (mean years 16 compared with mean years 13). Again, a chi-squared test showed this difference was significant at the 5% level (p < 0.05). There is no significant difference between those individuals who responded at time 3 and those who did not in terms of both total confidence scores and total attitude scores at both time 1 and time 2, although those who responded had slightly higher confidence levels (183.08 compared with 165.45 [total possible range 0–400]) and slightly more positive attitudes (31.64 compared with 32.29 [total possible range 14–70]) pre training.

Comments from the qualitative interviews supported the questionnaire findings and showed that participants felt the training addressed both attitudes and knowledge in a non-threatening way:

*'I think what, what I found very effective in it was that, and again I think this is why its good aimed at professional people, it ran through all the, it gives you some good background again which was, which refreshes you, which refreshes your knowledge base and it also refreshes your value base to a degree as well. So, I found that particularly useful and the fact that it did it in a non-threatening way and it just seemed to bring people onboard with the philosophy without, you know, without having to hammer it home as it were.' *(Trainee H193)

At time 2 people were asked to rate their satisfaction with the course across seven different elements (questionnaire available from authors). For each item the majority of people were entirely satisfied (60–92%); finding the course enjoyable, useful and relevant, with the right amount of detail. The lowest satisfaction scores related to people watching themselves on video in the 'therapist' role (item 5); however, only 6% of people reported this as being 'not at all' useful. In the qualitative interviews, participants also emphasised the positive value of both networking with colleagues and mutual learning:

*'I just think that fact that it was a varied group of people, you know they all, they all contributed something either through case histories or just, you know relating their own experiences of management risk, of assessing and managing risk.' *(Trainee H110)

### Impact on clinical practice

At time 3 respondents were asked in the written questionnaire about what elements of the STORM training modules had been of use in their everyday work. The responses to this question were categorised in two ways. Initially the responses were coded as to whether or not the participant had used, found useful or made a positive comment about a particular module, then the positive responses were further broken down into emergent themes. Of those who provided a response to these items the majority made a positive comment for both the risk assessment (90%) and the crisis management (84%) modules. Both modules 'provided reassurance' with 12% of responders commenting on this in relation to the risk assessment module and 16% of responders commenting on this in relation to the crisis management module. The responders spontaneous comments also indicated that the assessment module 'improved confidence' (15%) while the management module 'improved knowledge' (18%). The highest number of comments for any one theme related to 'asking more, and more direct questions' during 'risk assessment' (25%). The latter theme was highlighted in one of the qualitative interviews with a participant:

*'I had a farmer who was saying that he was suicidal and he had thought about hanging himself from one of the rafters... I asked him if he'd actually come to the point of picking exactly where in the barn it was going to happen and was there a particular rafter he'd even chosen, whereas I may not even have asked so specifically ....... the fact that it made me think about asking specific questions into how detailed is his, how close is he in actually formulating this plan, it definitely did help me.' *(Trainee H131)

Another participant commented on how she would not previously have seen through advice for a client to attend the doctor:

*'I thought maybe, maybe if they did have someone with them even to kind of support them getting to the doctor's or waiting for an appointment or simply getting them to stay in the doctor's surgery was actually the hardest thing because they were really so agitated that we had to wait for quite a while to be seen by a doctor and I think had I not been with them, I think they would have just walked out anyway. So, yeah, I don't think I would have done that before. I would have just said "oh I think you should see a doctor" and left it at that you know and gone on my way'*. (Trainee 191)

However, there was also a viewpoint from some experienced workers that *'a lot of it was what we were doing anyway, but just possibly to a higher standard' *(Trainee H172).

### Diffusion and adoption of the STORM intervention

Utilising the conceptual framework described by Greenhalgh and colleagues [29] we were able to identify the key stages and processes in diffusion and implementation of the intervention from the interview data. The STORM training intervention had not previously been used in Scotland. The Consultant in Public Health who was the local *opinion leader *[33], identified STORM as a possible training intervention from a search of the published literature:

'I went kind of thematically to the published literature which is not necessarily I think the way other folk would have pursued it

*I suppose, what I had in my head at that time was kind of a multi-level kind of training strategy, so I felt fairly clear about where the different bit of training probably fitted and even although some of them weren't actually available then I was pretty clear where they were going to go, so what we sold to the various committees was a kind of vision of what the overall training package would look like when the bits were available.' *(Consultant in Public Health)

What was envisaged encompassed separate suicide prevention training packages for the community level (ASIST; ) and health workers (STORM), with a Master's level training for specialist mental health staff. Some concerns were expressed about the local applicability of STORM, and so local workers took the opportunity to try out the intervention; providing opportunity for *trialability *(experimentation on a limited basis) and *observability *(benefits visible to intended adopters) [34]:

*'the key things I think were identifying a couple of folk to try the course... because although we were fairly positive about it, it was important that we had local staff that were comfortable with it... and the feedback was positive; the staff liked it, thought it was useful- they'd some concerns about some of the supporting materials which they felt didn't necessarily mesh with (a) Scotland and (b) with the comparatively rural services here. Apart from that they were happy with the skills part of it.' *(Consultant in Public Health)

Possible concerns were also expressed about the applicability of the training to experienced staff:

*'I'd had some concerns with the published work about the finding in the first or second paper that the specialist staff liked it but didn't necessarily benefit from it. So [on] one of the early courses actually we included some trained staff and the general view from the trainers was that in fact it probably did improve the skills of some of the trained staff. It kind of depended on who you were pitching it at essentially so that was pretty reassuring.' *(Consultant in Public Health)

The STORM team indicated that they were willing for potential adopters to modify the training to suit their own needs:

*'I think the fact there was some eventual willingness to revise some of the training materials for the local situation was very useful it certainly contrasted to an extent with [another training package] we were told we could change absolutely nothing under any circumstances ...it felt to us I mean closer to a partnership and I suppose that made people feel more comfortable with it .... I mean we think we're the experts in what works in rural areas and our kind of rural is not the same as England's kind of rural'*. (Consultant in Public Health)

And dissemination within the system began, with organisation of the necessary resources and commissioning of the training of a group of facilitators to provide training across Highland Region.

### The dissemination process

If dissemination is *'active and planned efforts to persuade target groups to adopt an innovation' *[29], the most important part of this stage was engaging and training the facilitators who were to implement the training within the organisation. Their engagement was absolutely essential to the task. Identified facilitators were provided with the training necessary to run local STORM courses. The first of three groups of 4 Facilitators were trained in December 2003. Two other courses were commissioned in March and April 2004. In all, 12 facilitators were trained by GLG. Training took place in Inverness, using STORM Suicide Prevention Course Version 1. The four modules consist of Assessment, Crisis Management, Problem Solving and Crisis Prevention (see table 8). Each module follows the same structure of brief lecture covering the salient points, demonstration with video excerpts, rehearsal using role-play and videotaped role-play with group feedback and discussion. STORM Facilitator training in suicide prevention is a four-day course. All four modules of suicide prevention are covered in the first two days (familiarisation with the model as participants) followed by a further two days of consolidation, practice delivering the course and discussion (training in facilitation of the STORM modules). The course can be delivered in two formats, either four consecutive days or split into 2 two-day sessions over two-weeks. The first group opted for the split format of training, spread over a three-week period to accommodate previously booked holidays; a practice not endorsed ordinarily but agreed upon due to the urgency of the training. The following two groups received training in the four-day training format. Each Facilitator was provided with a package of STORM materials needed to deliver courses across Highland Region. Evaluation of outcomes (see above) was built into the training.

**Table 8:** The STORM training package

There are four modules:	• Assessment
	• Crisis management
	• Problem-solving
	• Crisis prevention
	Each module is flexible, and if necessary can be delivered in 2 hours
Educational methods used in each training session:	1. Brief lectures on background knowledge and the skills to be acquired and rehearsed
	2. Focused group discussion
	3. Video demonstration of skills by health care professionals
	4. Role-play (rehearsal of skills) in trios (professional-client-observer) and pairs (professional-client) using pre-prepared role-play scripts to facilitate the practice of specific microskills
	5. Video-feedback in small group setting of recorded role-played interviews carried out by course participants
	6. Discussion to consolidate learning; specifically the translation of skills learned into practice

The material can be modified in content for:	• Primary care teams
	• Mental health care staff
	• Accident and emergency staff

From the interviews with the facilitators a number of key factors in dissemination emerged. Financial support was available from the Choose Life initiative to purchase equipment required for training. One of the facilitators was funded to be a central coordinator and trainers were also funded for dedicated training time out of their usual jobs in order to implement the programme. This dedicated time and resources proved to be invaluable:

*'The coordinator for 'Choose Life' strategy here has paid for one day of my time on a fairly consistent basis so almost a day a week I've had to be able to prepare and organize and deliver and that's been extremely helpful. In fact I couldn't have done it without that, it would have been impossible.' *(Facilitator 5@T1)

### Implementation of the training

Some of the factors that have already been mentioned in association with diffusion, adoption and dissemination (for example, leadership and administrative support) were important in supporting the implementation process. However the motivation, capacity and competence of individual workers was also of key importance [29]. A devolved, flexible and supportive managerial and administrative structure, provided both co-ordination and administrative support functions for the implementation process. Furthermore, this structure supported the roll-out of the training by the facilitators with regular peer support and training meetings:

*'Getting the trainers together to review their own work, throwing up different issues and problems that might be unique to a particular area.' *(Facilitator 5@T1)

*'We get everybody back in to practicing with all the technical equipment, the computers, the laptops, the projectors, the DVD players.' *(Facilitator 6@T2)

Facilitators also found it particularly helpful to work together within training:

*'It's much better co-facilitating for a number of reasons firstly it gives you two trainers and you can bounce off each other...also if you get somebody in the audience who becomes too distressed then one trainer can deal with that whilst the other one continues with the course for example we had a lady who was very distressed because she had a daughter who had committed suicide and we really had to take her out of the group and spend a lot of time with her.' *(Facilitator 27@T2)

Providing clinical cover for trainers sometimes put a strain on colleagues:

*'If I was to do it too often good nature might start to wear thin.' *(Facilitator 25@T2)

However, the major obstacles that the facilitators had to overcome came from the health and social care organisations themselves, who sometimes posed resistance to the process at managerial level, despite senior management support:

*'We have had indications that in at least one area the managers are trying to get people who attend to sign a form saying that if they leave within two years they'll pay the time back, which we're kind of disturbed about and we don't know how far out does it go, and is it the reason for health visitors not attending the course.' *(Facilitator 27@T1)

But this was by no means a universal problem:

*'My manager has not expressed any concerns and I believe that the general management attitude is supportive.' *(Facilitator 27@T1)

*'We've got definitive support from their line managers now to release them, which was something that was a sticking point in the past, mainly because we delivered a course to the line managers and convinced them.' *(Choose Life Coordinator)

The most challenging parts of the training were managing the role-play and video feedback, which professionals were sometimes reluctant to engage in:

*'A psychiatrist would you believe, wouldn't do it, you know the people that you would imagine would be most comfortable with their skills apparently appear to be the most ill at ease in displaying them.' *(Facilitator 8@T1)

But this element was also seen as one of the strengths of the method:

*'They're brought up short you know through being videotaped and observed .. it's the difference between what they believe they're asking...and seeing and ... what the actuality is.' *(Choose Life Coordinator)

The facilitators differed in how much they felt able to adapt the materials to their own training style and requirements:

*'I think it gives you a framework within which you work and I follow through the sequence of the overheads and we look at some of the tapes but I probably use the tapes less than I did when I originally started and I want people to do their own work in the experiential learning part, so I think we're all doing it slightly differently.' *(Facilitator 5@T1).

*'I still find it quite difficult delivering someone else's script almost, I know its not scripted as such in that you've got the presentation and then obviously what words you put round it are individual.' *(Facilitator 21@T1)

However, there was a sense of the utility and value of the product despite these problems:

*'STORM talks about levels of risk and although that's been an interesting and controversial area for STORM trainers I think it's something you have to kind of explore a bit. The reality that most clinicians might face is that they'll get ten people turning up saying their suicidal, what they have to identify is which of these people really are at risk and which of them are just in a state of crisis which is going to pass relatively quickly and probably won't need any, any major issues around personal safety. That's a difficultly for professionals identifying maybe the slightly intoxicated Friday night person who turns up at A+E saying 'I'm terribly upset, I've split up from my girlfriend, I'm going to kill myself' as opposed to the other person who may have been thinking about it for a long time and has got that much more structured plan.' *(Facilitator 5@T2)

### Routinization

Greenhalgh and her colleagues [29] do not distinguish implementation from the process of 'routinization', thus giving the impression that it is a part of the same process. However, little research has been undertaken to fully understand how an innovation becomes standard practice [29,34]. What evidence does exist suggests that successful implementation does not necessarily account for routinization [35-37]. According to Yin [32] 'routinization', which we understand to mean how an intervention becomes 'standard practice':

*'may follow a series of stages: an improvisation stage, an expansion stage, and a disappearance stage (disappearance means that the innovative practice continues, but is no longer regarded as new)*(p22).

When we followed up the facilitators there was some evidence of the intervention becoming no longer regarded as new, but incorporated into standard practice:

*'We're getting a consistent approach to risk assessment and risk management now in the field and that is as I say rippling out into other client areas and I mean so we have a single risk assessment strategy and policy in Highland now.' *(Choose Life Coordinator)

STORM had also been incorporated into nurse training on a University Course:

*'It's almost become a kind of event now that we deliver it to the students in semester seven I think it is, it's almost, it's almost as if it's become part of the programme.' *(Facilitator 5@T2)

The 'almost' here is interesting, giving the sense that training was still 'extra' to the normal curriculum of activities for professionals and *'bolted on'*, competing with other demands currently in fashion, not embedded in it.

STORM training seemed to be having a wider impact on organisations, aside from changes in individuals' clinical practice. Participants were motivated to review their departmental suicide prevention policies and procedures and, in some cases, attempt to improve them. However, in the later interviews it was evident that it was difficult to maintain the earlier momentum of a new intervention:

*'I think we're in their third year now so people are kind of, a little bit running out of steam.' *(Facilitator 5@T2 November 2005)

*'I think there's some challenges in just around 'how do we keep this going?" *(Facilitator 20@T2 February 2006)

Although some reduction in training enthusiasm was reported by facilitators, as time went on participants became more eager to attend training and demand for training grew once more from colleagues of those who had attended:

*'Initially it was potentially a pilot but as words got out and word of mouths gone round etc. just how good the course is people are clamoring for it...'We're always oversubscribed for the courses that's why we're kind of doubling the number of courses we're going to deliver...' *(Choose Life Coordinator commenting on progress to date in February 2008)

## Conclusion

Utilizing primarily the model described by Greenhalgh and her colleagues [29] we have described in detail the key stages of diffusion, dissemination and implementation of a training intervention for suicide prevention, the STORM programme, as part of the multifaceted Choose Life initiative [11] in the Highland Region of Scotland. Key factors in the process were the presence of a champion or local opinion leader ('The Adopter') who supported and directed the intervention, local adaptation of the materials ('Linkage'), commissioning of a group of facilitators who were provided with financial and administrative support, dedicated time to provide the training and regular peer-support ('The implementation process' and 'System readiness for innovation'). We have also described the impact of training on participants (excluding acquisition of clinical skills which we did not assess on this occasion), which was comparable with our previous studies.

We can conclude that the features that contributed to the success of STORM in this context were *both related to the context *(the multi-dimensional support provided from the host organisation and the favourable policy environment) *and the intervention *(openness to local adaptation, clinical relevance and utility), and these appeared to interact in a dynamic and recursive relationship. The initial drive from the champion and supported facilitators (context) being followed by increase in demand for training if it was perceived as relevant and useful (intervention); necessitating organisational support to meet demand and implement policy changes (context) and resulting in further change to and improvement of the training (intervention) and so on. Applicability and acceptance of the innovation was evidenced by way of a change in staff attitudes, confidence and reported impact on clinical skills. Networking across organizational structures, as a result of multidisciplinary training sessions, helped to formalise the experience and communicate to other practitioners the innovation's usefulness.

We acknowledge a number of limitations of this study. We relied largely on quantitative data collected immediately after the training, by those who had indeed delivered it. Those who responded at six months may well have been biased towards individuals with a more positive view of the intervention. We did not capture the views of those who did not participate in training at all, and only have the facilitators' views of the challenges in recruiting staff for training. Those whom we were able to contact for interview may also have been favourably biased towards the STORM intervention. We also acknowledge that, given that the dissemination was led by a healthcare agency, we have not considered the wider implementation of STORM across and within other agencies.

From the perspective of this evaluation, the Greenhalgh et al. model [29] has a number of strengths. It provided us with a pragmatic model and framework for the analysis of our data that is evidence based and makes logical sense. However it also has some shortcomings. It does not adequately attend to the process of routinization which we believe is an important and distinct process. It is also more concerned with understanding what elements are necessary within diffusion, dissemination and implementation processes rather than how those elements interact to be of importance to the process. The model does not focus on the work that, for example, a champion or local opinion leader actually has to do in order to facilitate this recursive process. For this we consider that Normalisation Theory as described by May [35], which specifically considers the 'workability' of interventions in everyday practice, would provide a superior framework for investigating how interventions can be incorporated into health and social care systems; unlike the model described by Greenhalgh and her colleagues [29], the Normalisation Process Model specifically addresses incorporation into routine practice, but describes a more complex process than the stepwise 'routinization' described by Yin, addressing the work that has to be done to achieve 'normalisation' [32]. This may be problematic and meet particular barriers late into the process of implementation when the initial enthusiasm and support, particularly at a managerial and policy level, has waned or specific project related funding has ceased. What is unclear therefore is whether 'routinization' inevitably occurs if the process we have described above is effectively managed, and *what is required, from whom*, to make that management 'effective', in other words what are the *mediating practices *of these recursive relationships? Exploration of this process requires a lengthy commitment on the part of both organisations and researchers to continue in the collection of data through different stages of this process. This will be investigated in a study of the wider implementation of STORM training throughout Scotland.

## Competing interests

The authors are all involved in the development and dissemination of STORM which is a not-for-profit venture hosted by the University of Manchester.

## Authors' contributions

The project was conceived by LG and GLG. Data collection was carried out by RH with assistance from GLG. All the authors participated in the analysis of the data. The paper was drafted by LG and modified and approved by GLG and RH.

## Pre-publication history

The pre-publication history for this paper can be accessed here:


